# Discussions about preventive services: a qualitative study

**DOI:** 10.1186/1471-2296-9-49

**Published:** 2008-09-03

**Authors:** Karen E Lasser, Bridget Kelly, Jan Maier, Jennifer Murillo, Sonia Hoover, Karen Isenberg, Deborah Osber, Natasha Pilkauskas, Bayo C Willis, James Hersey

**Affiliations:** 1 Department of Medicine, Cambridge Health Alliance and Harvard Medical School, Cambridge, MA, USA; 2 RTI International Inc, Washington DC and Waltham, MA, USA; 3 National Center for Immunization and Respiratory Diseases, Centers for Disease Control and Prevention, Atlanta, GA, USA

## Abstract

**Background:**

Elderly minority patients are less likely to receive influenza vaccination and colorectal cancer screening than are other patients. Communication between primary care providers (PCPs) and patients may affect service receipt.

**Methods:**

Encounters between 7 PCPs and 18 elderly patients were observed and audiotaped at 2 community health centers. Three investigators coded transcribed audiotapes and field notes. We used qualitative analysis to identify specific potential barriers to completion of preventive services and to highlight examples of how physicians used patient-centered communication and other facilitation strategies to overcome those barriers.

**Results:**

Sharing of power and responsibility, the use of empathy, and treating the patient like a person were all important communication strategies which seemed to help address barriers to vaccination and colonoscopy. Other potential facilitators of receipt of influenza vaccine included (1) cultural competence, (2) PCP introduction of the discussion, (3) persistence of the PCP (revisiting the topic throughout the visit), (4) rapport and trust between the patient and PCP, and (5) PCP vaccination of the patient. PCP persistence as well as rapport and trust also appeared to facilitate receipt of colorectal cancer screening.

**Conclusion:**

Several communications strategies appeared to facilitate PCP communications with older patients to promote acceptance of flu vaccination and colorectal cancer screening. These strategies should be studied with larger samples to determine which are most predictive of compliance with prevention recommendations.

## Background

Influenza and colorectal cancer are preventable diseases that result in substantial morbidity and mortality. Influenza and its complications contribute to an estimated 250,000 to 500,000 deaths worldwide each year [1]. Each year, 1 million new cases of colorectal cancer are diagnosed globally and more than 500,000 people die from the disease [2]. Even in the United States, despite the existence of effective means to prevent influenza [3], only 65% of adults aged 65 and older report receiving influenza vaccination during the previous 12 months [4]. Similarly, despite the availability of effective screening modalities [5-7], a large proportion of Americans are not being screened for colorectal cancer [8,9].

As a result, an important priority of research on these health promotion behaviors must be to identify specific barriers which prevent older adults from engaging in them. The Precede/Proceed Model, developed by Larry Green and Marshall Kreuter [10], provides a valuable theoretical framework for considering such barriers because it reminds us that we must think beyond individual-level factors such as lack of knowledge and consider environmental contributors as well. The Precede/Proceed model defines three types of factors which influence behavior: predisposing, enabling, and reinforcing [10]. Predisposing factors are characteristics that motivate a person to engage in behavior. These can include beliefs, attitudes, or knowledge or demographic background factors thought to impact the likelihood of engaging in the behavior. Enabling factors include characteristics of the environment that facilitate the behavior, as well as skills or resources, such as health insurance or ease of transportation, which make it possible to engage in the behavior. Reinforcing factors are defined as rewards or punishments which follow the behavior or are anticipated as a consequence of the behavior [10]. Expectations about the support of friends and family for a behavior are often viewed as important reinforcing factors.

Partially because some of these barriers are likely to be more prevalent for members of some racial and ethnic groups than for others, disparities in receipt of both influenza vaccines and colorectal cancer screening tests have been found between nonwhites and whites in the United States [11-15]. Such disparities are only partially explained by differences in access to care [16], nor do they seem to be fully explained by patient beliefs [17-20] or provider attitudes [21]. Rather, Kilbourne and colleagues suggest that a key component may lie in the clinical encounter between patients and providers [22]. This article explores the potential of studying patient-provider encounters among older adults.

The Institute of Medicine has suggested that various features of the patient-physician relationship may contribute to disparities [23]. Specific elements of the interaction which may play a role in the existence of disparities are the provider's skills or lack thereof in cultural competence and in communication [22]. For example, if providers fail to tailor messages appropriately about health promotion or disease prevention, that can lead to lack of adherence to the prescribed behavior [22].

As for provider communication, researchers have found "patient-centered communication" important to achieving better patient recall of information, treatment adherence, satisfaction with care, and health outcomes [24]. Patient-centered communication is exemplified by encounters in which "the patient's point of view is actively sought by the physician" [25].

Researchers seem to agree on three key areas or components of patient-centered communication: (1) developing an understanding of the patient as a person, (2) conveying empathy, and (3) finding common ground regarding treatment and goals of care or, in this case, prevention [26]. Understanding the patient as a person means reaching beyond the physical symptoms to understand other important aspects of the person's lifestyle or context [27]. The idea of empathy, or the patient's perception of the doctor as caring and sensitive, is thought to be important to compliance with recommended behaviors [27].

The third construct, finding common ground, is described as sharing decision-making responsibility between the physician and patient or empowering the patient. Sharing power or responsibility allows patients to play a more active role in decisions related to their care. This idea is in line with Williams, Frankel, Campbell and Deci, who write that "relationship-centered care," is related to Self Determination Theory because one of its central components is autonomy support or "interacting with patients by taking full account of their perspectives, affording choice, offering information, encouraging self-initiation, providing a rationale for recommended actions, and accepting the patients' decisions" [28].

While a number of prior studies have audiotaped and observed patient-primary care provider (PCP) encounters [29-34], we are unaware of prior studies that have directly observed and analyzed in qualitative terms how PCPs and patients discuss prevention of colorectal cancer or influenza. We chose to study two preventive services in tandem to compare and contrast barriers of each and to explore how patient-centered communication and other facilitators were used to overcome those barriers.

To understand how aspects of the patient-PCP communication affect receipt of these preventive services, we performed a qualitative study, observing and audiotaping medical visits at two community health centers. We also studied systems of care, such as whether staff routinely identified patients as needing a particular preventive service, or whether systems barriers such as a long wait time for a colonoscopy appointment prevented completion of services. Our primary objectives were (1) to describe the dialogue between PCPs and elderly patients about influenza vaccines and colorectal cancer screening and (2) to identify both potential barriers to and facilitators of completion of these preventive services (including all three types of factors specified by the Precede/Proceed model and specific strategies for patient-centered communication).

## Methods

We conducted an in-depth observational study of two urban community health centers in greater Boston. We initially identified five health centers with a high minority population over the age of 65; reasons that three of the five health centers did not participate included transition to an electronic medical record, changes in health center leadership, and physical damage to the health center from a car accident. Our study design involved administration of questionnaires, observation of office systems, and observation and audiotaping of clinical encounters. Trained research assistants performed these tasks. Two medical anthropologists assisted in developing the observation form. Two research assistants were bilingual in Spanish and in English; we trained four additional interpreters to assist with Spanish and Haitian Creole-speaking patients. We also conducted in-depth interviews with key informants at each health center (medical directors, nurse managers, and nurses) to provide a context of systems and community factors.

### Participants

In the fall of 2005, we recruited participants from two health centers. Research assistants approached a convenience sample of patients who presented to the health centers during the recruitment period (October to December 2005, the time of year when the influenza vaccine is available and most discussion about vaccination takes place). We informed patients that the purpose of the study was to "learn how doctors and nurses talk with older patients about their health," and offered patients a $10 cash incentive to participate. The research assistants asked whether the patients had received any of the following preventive services: a vision test in the past year, a hearing test in the past year, a pneumonia vaccine ever, and an influenza vaccine since August 2005. We deliberately asked about multiple preventive services in an effort to blind the study's primary objective, which was to observe discussions about the influenza vaccine and about colorectal cancer screening.

Patients who were aged 65 or older, who spoke English, Spanish, or Haitian Creole, and who had not received an influenza vaccine in the current year were eligible to participate. After determining the patient's eligibility for the study, the research assistants asked patients whether they would be willing to have their appointment observed and audiotaped. Study investigators also approached PCPs (physicians and nurse practitioners) at each site, obtaining their permission to have encounters observed and audiotaped. We told the PCPs that they were participating in a study to examine communication between patients and providers about preventive services. We audiotaped and observed a total of 18 clinical encounters involving 7 PCPs. Eleven patients refused to participate in the study, and 20 patients were ineligible to participate because they had already received the influenza vaccine. The Institutional Review Boards at Cambridge Health Alliance and at RTI International approved the study; all patients and providers gave written informed consent.

### Data collection

Prior to their visit, patients completed a brief survey that included questions about the purpose of the visit, health care use, risk factors for complications of influenza, and demographics. Following the visit, patients answered questions about their perceptions of the visit; their beliefs and attitudes about influenza vaccines, colorectal cancer screening, and mammography (women only); and additional demographic questions. PCPs completed a background questionnaire about their demographics. Research assistants set up a digital voice recorder in the exam room and remained in the exam room unless requested to leave by either the PCP or the patient. In only one encounter did the observer need to leave the room. The research assistant observed and made notes about the PCP/patient interaction, and stood behind a curtain during the physical examination. The research assistants received training to observe aspects of the encounter including physical contact between patient and PCP, use of hand gestures, eye direction, facial expressions, listening, interruptions, and level of comfort. One investigator (KEL) reviewed both paper and electronic medical records 9 months after the visit to determine whether preventive services (immunization for influenza and colorectal cancer screening) were completed. In the one case where the record did not provide the necessary data, a research assistant spoke to the patient by telephone to obtain further information.

### Analysis

We obtained verbatim transcriptions of all encounters, and identified all segments in which the providers and patients discussed either influenza immunization or colorectal cancer screening. For most of the Spanish-language encounters, a bilingual research assistant (JM) who observed the encounters both transcribed and translated the text of the encounter. We also analyzed detailed descriptive field notes that research assistants recorded during the observed encounters. An analysis team composed of a primary care physician-researcher (KEL), a nurse researcher (JEM), and a health behavior specialist (JH) read through all transcripts and field notes and discussed the details of each encounter. We identified potential barriers to and facilitators of completion of preventive services that emerged in these discussions, as well as specific patient-centered communication strategies. We reviewed and critiqued interim versions of the main barriers and facilitators in an iterative process. We present the barriers and facilitators upon which all three analysts and the study team agreed.

## Results

### Description of health centers and systems for promotion of preventive services

Health center 1, located in a city west of Boston, serves mainly African American and Haitian patients. This health center uses paper charts, does not use a flow sheet to track preventive services, and has no reminder system in place for preventive services. A nurse at this site educates patients about preventive services, particularly colorectal cancer screening (many patients, after seeing the gastroenterologist, have questions about how to complete the preparation for the test). However, most patients do not routinely see a nurse to discuss preventive services. During influenza season, staff post signs inside and outside the health center advertising influenza clinics. Patients are then able to walk in and obtain an influenza vaccine at the center without an appointment. We were only able to recruit two patients for the study at this site. Barriers to recruitment included a lack of availability of a Haitian Creole interpreter and a high number of patients who walk in for care without a previously scheduled appointment. We could not include patients who walked in because we were unable to anticipate research staff requirements (observer and interpreter) for these patients.

Health center 2, also located in a city west of Boston, serves a large Latino patient population. This health center uses an electronic medical record with an electronic flow sheet for tracking age-appropriate preventive services. The center staff called patients with diabetes (from a diabetes registry) to come in for an influenza vaccine. We recruited 16 patients for the study (both with and without diabetes) at this site; we suspect that our use of a bilingual Hispanic research assistant (JM) facilitated recruitment.

At each health center, both PCPs and nurses administered influenza vaccines to patients. Cost was not a barrier to receipt of the influenza vaccine at either health center. Structural factors impeded colorectal cancer screening efforts at both health centers: there was a long wait for routine colonoscopy appointments (approximately 9 months), and neither center had a system in place to track distribution and return of fecal occult blood testing (FOBT) cards.

### Patient-PCP encounters

We observed 18 unique patient visits to 7 different PCPs. Table 1 shows the demographic characteristics of the 18 patients. Most were female, nonwhite, Spanish-speaking, poor, and with a low level of education. The mean age of participants was 71.9, and all had some form of health insurance. PCPs were physicians and nurse practitioners, trained in either family medicine or in internal medicine. Most of the PCPs were nonwhite, spoke Spanish fluently (although none identified as being Hispanic), and had practiced at their respective health center for at least 6 years. The average patient-PCP encounter length was 24 minutes.

**Table 1:** Community Health Center Patient Characteristics (n = 18)

**Characteristics**	
Female (%)	77.8
Mean Age (se)	71.9 (7.8)
Race (%)	
White	22.2
Black	16.7
Mixed	27.8
Other	33.3
Hispanic or Latino origin (%)	72.2
Insurance (%)	
Medicare	77.8
Medicaid	16.7
Free Care	5.5
Language used in visit (%)	
English	27.8
Spanish	72.2
Education (%)	
< High School	66.7
High School diploma	11.1
Some higher education	22.2
Annual Income (%)	
< $10,000	44.4
$10,000–$14,999	5.6
$15,000–$19,999	16.7
$20,000–$25,000	11.1
Don't know/missing/refused	22.2

PCPs and patients discussed the influenza vaccine in 16 of 18 (88.9%) encounters. The influenza vaccine was not discussed in the following two situations: (1) in a visit that took place prior to the availability of the influenza vaccine and (2) in an urgent care visit for a complaint of a red eye. In most cases (14 of 16 [87.5%]), the PCP introduced the subject of influenza vaccination. In 13 of 16 encounters the PCP vaccinated the patients; in the remaining encounters, a nurse vaccinated one patient after the PCP visit, a second patient refused the vaccine, and a third patient was ill and needed to return for the vaccination. The latter patient did not return to the health center to be vaccinated. When we called this patient several months later, she reported that she was preparing to have knee surgery and was unable to return for her influenza vaccine because she had difficulty walking.

PCPs and patients discussed colorectal cancer screening in 8 of 18 (42%) encounters. In four of eight encounters, the patients were either out of the age range for screening (age > 80) [35] or had already been screened. In the remaining four patients, three were screened during the follow-up period (two patients completed colonoscopy and one patient completed FOBT cards). One patient (the same patient who did not return for the influenza vaccine) did not complete FOBT cards because she had been ill. She also assumed her colon was normal because she had had many tests prior to her knee surgery, and felt that if she had a colon problem those tests would have detected it.

### Use of patient-centered communication and other facilitation strategies to overcome barriers

Through direct observation of visits and analysis of transcripts of the audiotaped encounters, we identified examples of all three types of barriers described in the Precede/Proceed framework (predisposing, enabling, and reinforcing). Table 2 lists these potential barriers.

**Table 2:** Barriers identified from patient-provider encounters

Precede/Proceed construct	Barrier
Predisposing factors (beliefs, attitudes, knowledge, demographics, background)	Fear of becoming ill from influenza vaccine No symptoms of colorectal cancer
Reinforcing factors (rewards or punishments which follow the behavior or are anticipated as a consequence)	Anecdotes of negative experiences with influenza vaccine
Enabling factors (environmental factors, such as health insurance, cost, and structural barriers, such as ease of access to care)	Patient unable to receive influenza vaccine during visit due to acute illness Dependence on others for transportation makes return visits more difficult to schedule Misunderstanding/misinformation about cost of influenza vaccine Complexity of colorectal screening process Many topics covered in visits

We then identified ways in which patient-centered communication and other strategies (including cultural competence) were used to address barriers to acceptance of influenza vaccines and colorectal cancer screening. Table 3 provides specific examples from the patient encounters of patient-centered communication strategies and other facilitators.

**Table 3:** Examples of patient-centered communication strategies and other facilitators used by PCPs to address barriers

**Barrier**	**Example from transcript**	**Patient-centered communication strategy**	**Other facilitators**	**Outcome**
Lack of knowledge about CRC	**Example 1: 66-year-old, Spanish-speaking Hispanic male** PCP: I also would like to talk to you about the colonoscopy. Have you had it done in the past? Would you remember what it is about? PCP then explains colonoscopy. PCP: You can think about it, if you would like to have it done, this test, but it is possible. PCP returns to CRC discussion after giving shots. PCP: Okay, what do you think of the possibility of having done the colonoscopy test? P: It would be good, right? PCP tries to schedule GI appointment at best time for patient.	Shared power/common ground		GI appointment scheduled
				
Anecdotes of negative experiences with influenza vaccine	**Example 2: 66-year-old Spanish-speaking Hispanic woman from Puerto Rico** PCP: I don't know if you want to get the shot against the flu? P: Ay no! PCP: Why not? P: I have never gotten it before because I heard it gives people...My brother in law got it and he was in the hospital for more than a month with the flu, with fever, vomits, he got everything. 'Ay, cunada don't do it' (sister-in-law, don't get the flu shot!) so I never got it. No, no, I won't do it. PCP tries to convince patient that reaction is a very rare event, recommends strongly, gives patient a chance to think about it during the visit. Later in visit: PCP: And what have you thought about the shots? P: (laughed) Ay doctor, I am not frightened by the injection, I am afraid of the reaction, such as fever or something like it. PCP: Would you like to try it, the reactions are rare, but you are the one who has to make the decision. PCP negotiates which arm to apply shots, given that she has arthritis in one arm – decides shots should go in bad arm so will still have one good arm. PCP: Very well, congratulations!	Shared power/common ground	PCP initiates discussion of influenza vaccine Revisiting the topic throughout the encounter	Patient receives vaccine during exam
				
Mis-understanding/misinformation about cost of influenza vaccine	**Example 3: 66-year-old Spanish-speaking Hispanic man (Salvadoran)** PCP explores patient's reason for not getting flu shot: Patient doesn't think he will get the flu. Also, doesn't want shot because is worried will get billed for it (last year he received a bill for it, and for PCP visit, has Medicare only). PCP decides to change way will bill for visit; not as PE but for cholesterol and stomach problems. PCP: Ah...what else...if I can give you the shot without any charge, would you have done it today? P: Yes. Later in visit PCP assesses patient's literacy in English, gives Medicare website to patient (patient's son reads English and has Internet).		Cultural competence (assesses English literacy before giving patient written information) PCP addresses incorrect beliefs/mis-information	Influenza vaccine is given during exam
				
Dependence on others for transportation makes return visits more difficult to schedule	**Example 4: 66-year-old Spanish-speaking Hispanic woman (from Dominican Republic)** PCP tries to make appointment at a convenient time for patient. PCP: When do you prefer the appointment? P: In the afternoon. In the morning she is working (referring to her daughter sitting next to her). PCP: What time is good for you? (Asking patient's daughter.)		Adapting to each patient's needs Facilitates scheduling of colonoscopy	Colonoscopy was scheduled
				
Patient does not speak English	**Example 5: 69-year-old Spanish-speaking male from El Salvador** PCP is talking in Spanish to patient, but PCP doesn't speak fluent Spanish.		Cultural competence	Influenza vaccine given during appointment; CRC screening not discussed, but patient has GI appointment in 2 days for weight loss
				
No symptoms of colorectal cancer	**Example 6: 66-year-old Spanish-speaking Hispanic woman** PCP: There is one test you haven't done, this is a test called "colonoscopy," have you heard of this test? P: You told me last time, you asked me to think about it, but... PCP: What did you think? You didn't like the idea. P: I don't like the idea. I imagine it is because I am feeling fine, maybe because I think illness gives you signs. PCP: The problem is that illness gives you signs when it is too late, and we have found that the way of finding out about it when there is still a cure for it, and this is the main purpose of this test. If you don't want to have this test done, there is another way of doing it, an easy way, I don't know if you have seen our cards, we will do this test every year. This is another way, it is not as good as the colonoscopy, but it is a way to do an evaluation, if you wish we can do it [FOBT]. In the lab you will get the cards and take them home with an envelope to send them back.	Shared power/common ground	Revisiting the topic between encounters	Patient does not return FOBT cards. When called several months later, she reported that she did not complete the cards because she has been ill. She also assumed her colon was normal because she had had many tests prior to her recent knee surgery and felt that if she had a colon problem those tests would have detected it.
				
No specific barriers (communication strategies used in normal course of visit)	**Example 7: 87-year-old Spanish-speaking Hispanic woman (from Colombia)** (Patient has lung, heart conditions.) Observer asked to leave room, tape turned off at one point. Patient hugged PCP, was crying. PCP not rushed at all, took her time. PCP very friendly toward patient, paid attention, listened carefully.	Empathy		Patient did get flu vaccine during exam; not in age range for colonoscopy
				
	**Example 8: 66-year-old Spanish-speaking Hispanic woman from Dominican Republic (same patient as example 4)** PCP: ...after you had the surgery, this is nothing. (Trying to give comfort to patient while applying the shots.) P: These shots hurt a lot: I think they make them for horses...I don't think you ever had one doctor, you should have one. (Laughed) PCP: Yes, yes, I did it already. (Laughed.) Somebody else gave it for me. (Laughed)	Empathy		

Sharing of power and responsibility was the most frequently used patient-centered communication strategy (see Table 3). In the first example, the provider brings up the topic of colonoscopy and asks the patient to think about it: PCP: "I also would like to talk to you about the colonoscopy. Have you had it done in the past?" PCP explains colonoscopy. "You can think about it, if you would like to have it done, this test, but it is possible." The provider returns to the topic after vaccinating the patient. "Okay, what do you think of the possibility of having done the colonoscopy test?" In this case, the provider is not telling the patient what to do, or even strongly recommending it, but merely presenting the information and asking the patient to consider it. The power to make the decision is left to the patient. When the patient decides it would be a good idea, the provider assists in scheduling the test at a time that would be convenient for the patient.

Example 2 demonstrates how a PCP is able to convince a patient, initially reluctant to have an influenza vaccine, to receive the vaccine by the end of the visit. The PCP uses several tools to facilitate the patient's acceptance of the vaccine: he or she revisits the topic throughout the encounter, giving the patient an opportunity to think about it, and empowers the patient by allowing her to choose which arm for the injection.

In another example (Example 6) the physician uses these same strategies of shared power and revisiting the topic multiple times to try to convince a patient to have the colonoscopy. In this example, the physician asks the patient, "There is one test you haven't done, this is a test called 'colonoscopy,' have you heard of this test?" The patient responds, "You told me last time, you asked me to think about it, but..." PCP: "What did you think? You didn't like the idea." P: "I don't like the idea. I imagine it is because I am feeling fine, maybe because I think illness gives you signs."

The physician clears up the misinformation by explaining that often when signs appear it is too late for early detection and cure, and that this is why the screening is important. But sensing that the patient is unconvinced, the physician goes on to explain that another alternative is FOBT cards. The physician is successful in convincing the patient at least to agree to take home FOBT cards. However, the patient does not return the cards to the office (see Table 3).

#### Empathy

An example of empathy occurred when one patient expressed how painful the vaccinations (one for flu and one for pneumonia) were (example 8). She said to the doctor, "These shots hurt a lot: I think they make them for horses...I don't think you ever had one doctor, you should have one." The PCP responded that indeed she had received the vaccinations, which let the patient know that she could relate to her pain.

#### Patient as person

Although it did not appear to be relevant to a specific preventive service barrier, one example (not in the table) of the "patient as person" construct occurred when a provider acknowledged a patient's upcoming vacation before introducing the topic of vaccination, saying, "Well, you just want to go Miami! Okay, one thing that you need is to have the flu shot given..." The results of treating a patient as a person were often observed in the high five with which a provider greeted an 83-year-old African-American patient, and the way in which this relationship helped a patient to overcome fear of immunization.

#### Cultural competence

There were several obvious examples in which cultural competence played a role. In one case, even though a provider was not fluent in Spanish, she spoke Spanish to her patient during the visit (Example 5). In another, the physician assessed the patient's English literacy before providing an English version of some Medicare information regarding the cost of the vaccine (Example 3).

#### Other facilitators

There were also several instances of a strong bond or relationship between patients and providers. In some examples, they hugged, gave each other high five or even expressed feelings of love for each other. In one example, the patient brought the provider a gift.

In another example (a 66-year-old Spanish-speaking patient), while the physician was out of the room, the patient was speaking to her daughter about possibly changing health centers on the advice of her sisters. The patient related that her sisters say to her, "It's like you got married with that doctor." Describing her 20-year relationship with the PCP, the patient said that her PCP loved her and has helped her a lot, and is like a family member to her. At the end of the visit, the patient says to her PCP, "I love you very much," and the two hug.

Our observation of a trusting relationship between PCP and patient was corroborated by the fact that in the post-visit debriefing questionnaire, all of the patients who completed the questionnaire (17 of 18) strongly agreed with the statement "All in all, I have complete trust in [PCP name]."

## Discussion

In this qualitative study, we observed that the following factors appeared to facilitate receipt of an influenza vaccine: patient-centered communication strategies, including shared power and responsibility, empathy and treating the patient as a person, cultural competence, PCP introduction of the influenza vaccine discussion, PCP vaccination of the patient, persistence of the PCP (revisiting the topic throughout the visit), and strong rapport and trust between the patient and PCP. We noted significant barriers to receipt of influenza vaccines: (1) acute viral illness (where the illness was perceived to be a contraindication to vaccination), and the patient had to postpone his or her influenza vaccine, requiring another trip to the health center; and (2) an urgent care visit for an acute complaint (as opposed to a routine health care maintenance visit) where preventive services were not discussed. Similar to the case of influenza vaccines, we found that patient-centered communication, the PCP's persistence, and strong rapport and trust between patient and PCP seemed to facilitate completion of colorectal cancer screening. Additional potential facilitators of colorectal cancer screening included the presence of someone else at the visit with the patient, and the PCP's assistance with scheduling. Barriers to colorectal cancer screening included (1) lack of symptoms suggesting a problem with the colon and (2) acute illness that made it difficult for a patient to return the FOBT cards to the health center.

Our observation that most PCPs discussed influenza vaccination with their patients appears to contradict prior studies that have shown much higher "missed opportunities to vaccinate" [36]. We suspect that our finding may reflect observation bias: because the PCPs knew we were observing discussions of preventive services, they may have been more likely to discuss vaccination. Our observation that trust between PCP and patient seemed to be associated with high use of recommended preventive services is consistent with prior studies [37]. Why did we observe such a high level of trust between the PCPs and their patients? It is possible that patients who agree to be observed may be more trusting than other patients. It is also possible that the population we studied, mostly Hispanic patients from El Salvador, Guatemala, Colombia, the Dominican Republic, and Puerto Rico, are particularly trusting of their PCPs. Among diverse populations of Hispanic patients, Mouton and Villa [38] have described a cultural phenomenon known as *personalismo*. *Personalismo *is an "inclination to relate [to] and trust individuals as opposed to systems or organizations." Such a level of trust was exemplified by the touching and hugging we observed, as well as by the verbal expression of mutual love and appreciation between patients and their PCPs. Finally, we observed a high level of race and language concordance between patients and PCPs. It is possible that such concordance, coupled with the fact that most of the PCPs had worked at their respective health centers for many years, contributed to the high level of trust we observed.

We observed a number of instances in which providers attempted to share the responsibility or power of decision making by providing the patient with the information about the preventive measure and then giving him or her time to decide whether to have the vaccine or schedule the colonoscopy. Phrases such as, "it's your decision" or "what have you decided?" were common. Stewart et al. found that interactions which scored high on patient-centeredness were actually associated with better emotional health 2 months later [25]. Other research has found that patients are more satisfied when interactions are patient-centered [39]. Thus, they may be more likely to adhere to recommendations that are patient-centered.

PCPs went "above and beyond" their usual responsibilities when they vaccinated patients during the encounter, and when they helped patients to schedule appointments. Given that PCPs report lack of time and a large number of preventive health issues they must address [40], having non-physician members of the health care team perform these tasks might enable PCPs to address other issues. Yet PCP discussion of preventive health services conveys a credibility and importance that can be particularly motivating to patients [41,42]. Moreover, several studies report that even busy physicians are able to talk to their patients about receiving adult immunizations [43]. We also observed that PCPs tailored their approach to discussing preventive services to the unique circumstances of each patient, demonstrating the practice of the "art" of medicine.

Our study is limited by the fact that we observed only a small number of patient-PCP encounters in two urban health centers. Among these encounters, we observed only four discussions of colorectal cancer screening in patients who were eligible for such screening. In addition, we do not have demographic data on patients who refused or were ineligible to participate. Thus, it is unclear whether this sample of patients is representative of patients engaged in primary care in Boston-area community health centers. Similarly, we do not know whether the practices of the observed PCPs (such as helping patients to schedule appointments or vaccinating patients) are representative of all PCPs who practice in community health centers. Our study required that an observer be present while patients were treated. This could have had a large impact on the conversation between doctor and patient. Another limitation was that we provided a cash incentive to participate. This may have influenced the validity of the information provided on the patient surveys. Due to study logistics, we were not able to include patients who walked in at health center 1. Thus the two patients we recruited at that site may not be representative of patients at that health center.

## Conclusion

Though not without limitations, our study is unprecedented. It provides valuable observations about how PCPs use patient-centered communication strategies to complete preventive services in disadvantaged elderly patients seen at community health centers. We observed that most influenza vaccines were given during the exam by the PCP and that the majority of the PCPs knew their patients for long periods and/or had established trusting relationships with them. During the observed exams, many PCPs were able to take the time to revisit preventive issues several times. In addition, in some cases, PCPs were also able to empower patients, to empathize with them, to correct misinformation, and to provide assistance in arranging follow-up. All these factors appear to influence the completion of influenza vaccination, and some of them may also impact the completion of colorectal cancer screening. Such observations warrant further study in a larger sample of patients, and may help to inform the design of interventions to increase rates of influenza vaccination and colorectal cancer screening in patients seen at community health centers. This study identified a number of potential barriers to these two screening behaviors and examples of how providers used patient-centered and culturally competent communication to address them. A larger study might include these measures to determine which are most predictive of compliance. Such research would help to illuminate which factors physicians should focus on in a time-limited appointment and which strategies are most effective in helping to promote these prevention strategies in minorities and, thus, help to reduce disparities.

## Competing interests

The authors declare that they have no competing interests.

## Authors' contributions

KL, co-principal investigator, led clinic recruitment, led analysis, and wrote and edited drafts of the manuscript. BK participated in analysis and drafting of the manuscript. JEM, study coordinator, oversaw training, patient recruitment, and data collection. JM coordinated data collection in one clinic and translated Spanish-language interviews into English. SH, KI, DO, and NP participated in data collection and analysis of results. BW participated in the initial study design and interpretation of findings. JH, the principal investigator, led study design and participated in the analysis and writing of this manuscript. All authors read and approved the final manuscript.

## Pre-publication history

The pre-publication history for this paper can be accessed here:


